# The role of oligodendrocyte precursor cells expressing the GPR17 receptor in brain remodeling after stroke

**DOI:** 10.1038/cddis.2017.256

**Published:** 2017-06-08

**Authors:** Elisabetta Bonfanti, Paolo Gelosa, Marta Fumagalli, Leda Dimou, Francesca Viganò, Elena Tremoli, Mauro Cimino, Luigi Sironi, Maria P Abbracchio

**Affiliations:** 1 Department of Pharmacological and Biomolecular Sciences, University of Milan, Milan, Italy; 2 Centro Cardiologico Monzino, Milan, Italy; 3 Physiological Genomics, Biomedical Center, Ludwig-Maximilians University, Munich, Germany; 4 Department of Biomolecular Sciences, University of Urbino, Urbino, Italy

## Abstract

Following stroke-induced neuronal damage, quiescent oligodendrocyte precursors (OPCs) are activated to proliferate and later to differentiate to myelin-producing cells. GPR17, a receptor transiently expressed on early OPCs, has emerged as a target to implement stroke repair through stimulation of OPC maturation. However, being GPR17 completely downregulated in myelin-producing oligodendrocytes, its actual role in determining the final fate of OPCs after cerebral ischemia is still uncertain. Here, to univocally define the spatiotemporal changes and final fate of GPR17-expressing OPCs, we induced ischemia by middle cerebral artery occlusion (MCAo) in reporter GPR17iCreER^T2^:CAG-eGreen florescent protein (GFP) mice, in which, upon tamoxifen treatment, cells expressing GPR17 become green and traceable for their entire life. Starting from 3 days and up to 2 weeks after MCAo, GFP^+^ cells markedly accumulated in regions surrounding the ischemic lesion; several of them proliferated, as shown by co-labeling of the DNA synthesis marker 5-Bromo-2′-deoxyuridine (BrdU). Almost all GFP^+^/BrdU^+^ cells expressed the OPC early marker neural/glial antigen 2 (NG2), indicating that they were still precursors. Accumulation of GFP^+^ cells was also because of OPC recruitment from surrounding areas, as suggested *in vivo* by acquisition of typical features of migrating OPCs, shown *in vitro* in presence of the chemoattractant PDGF-AA and confirmed by transplantation of GFP^+^-OPCs in wild-type MCAo mice. Eight weeks after MCAo, only some of these precociously recruited cells had undergone maturation as shown by NG2 loss and acquisition of mature myelinating markers like GSTpi. A pool of recruited GFP^+^-OPCs was kept at a precursor stage to likely make it available for further insults. Thus, very early after ischemia, GFP^+^-OPCs proliferate and migrate toward the lesion; however, most of these cells remain undifferentiated, suggesting functional roles other than myelination.

Neurological and neurodegenerative disorders are characterized by extensive loss of myelin sheath and defective remyelination.^1^ In the central nervous system (CNS), myelin is produced by oligodendrocytes, which originate from oligodendrocyte precursors (OPCs) expressing the neural/glial antigen 2 (NG2). The proliferation and differentiation of OPCs are greatly increased after brain damage in the region adjacent to the ischemic ‘core’, when they contribute to remyelination.^2^ However, despite injury-induced OPC activation, damage also progresses at later phases after ischemia, indicating that the capability of OPCs to maturate to new myelinating oligodendrocytes is only partially successful. Interestingly, the presence of recruited immature OPCs is documented at the border of the lesion even at later times after injury, suggesting that either their differentiation is blocked at the pre-oligodendrocytes stage, or that these cells exert additional roles besides differentiation to myelinating cells.

Recent data have highlighted the GPR17 receptor as a potential target to implement repair and remyelination under neurodegenerative conditions.^3, 4, 5^ GPR17 is already present in early NG2^+^-OPCs, is induced in differentiating cells up to the immature oligodendroglial stage, and is then progressively downregulated to allow cells completing maturation.^6, 7^

Interestingly, GPR17 expression is increased after traumatic brain injury in patients,^8^ after brain ischemia in mice^4^ and in several animal models of CNS injury.^5, 9, 10, 11^ However, the process underlying the accumulation of GPR17^+^ cells at the site of ischemic lesions,^4, 12^ the rate and kinetics of their maturation and their final fate are still largely unknown. Owing to the only transient expression of GPR17 that completely disappears before cells reach terminal maturation, it has been difficult to univocally follow *in vivo* the destiny and role of GPR17-expressing OPCs. To solve this problem, in this study we induced stroke by permanent middle cerebral artery occlusion (MCAo) in GPR17iCreER^T2^:CAG-eGreen florescent protein (GFP) transgenic mice, the first fluorescent reporter mouse line for GPR17 fate-mapping studies. In these mice, upon tamoxifen administration, enhanced GFP is expressed in OPCs where the GPR17 promoter is active, without affecting the physiological expression and function of the receptor. In this way, all cells expressing GPR17 at the time of tamoxifen administration (and their progeny) become fluorescent and can be easily visualized by fluorescence microscopy throughout their life.^10^

## Results

### After MCAo, GFP^+^-OPCs are rapidly recruited to the regions surrounding the ischemic lesion

Previous data on rodents indicate that, at early times after MCAo, GPR17^+^-OPCs start accumulating in the peri-lesion area, suggesting a role of these cells in reparative/regenerative processes.^10, 13^ However, no data on the fate of these cells at longer times after MCAo are available. To shed light on this issue, we induced MCAo in the conditional GPR17iCreER^T2^:CAG-eGFP mouse line for fate-mapping studies^10^ (Supplementary Figure 1).

**Figure 1:**
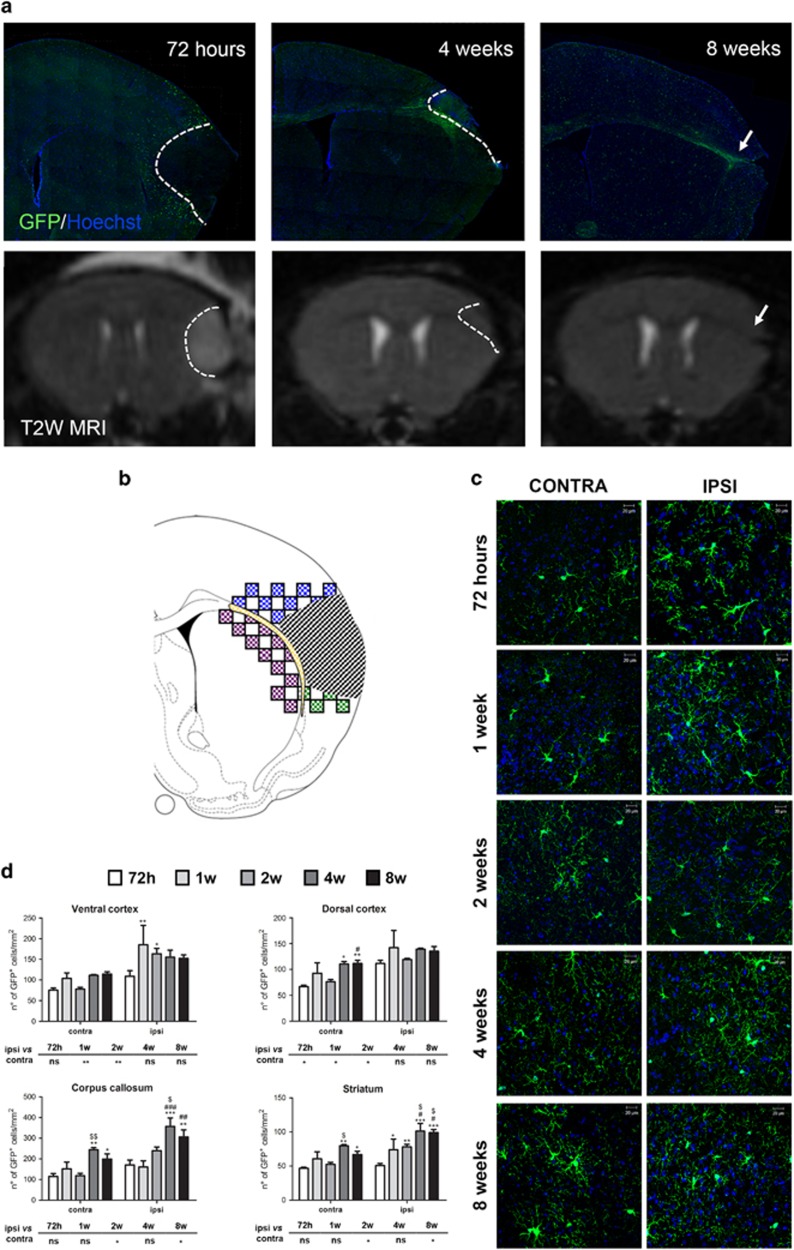
GFP^+^ cells are recruited in regions surrounding the ischemic lesion. (**a**) Representative image of GFP immunofluorescence in ischemic brain slices from GPR17iCreER^T2^:CAG-eGFP mice 72 h, 4 and 8 weeks after MCAo. Representative T2W brain images of the same mouse at the same slice level for each time point. At 72 h, the dashed line delimitates the vasogenic edema, which is not visible at 4 weeks after MCAo (the dashed line delimitates the atrophic damage). Arrow indicates the elongation of corpus callosum and the cortical atrophy at 8 weeks after MCAo. (**b**) Drawing showing the ischemic lesion (dashed area) and the sampled fields in the selected ROIs: dorsal cortex (blue), ventral cortex (green), corpus callosum, cc (yellow) and corpus striatum (purple). (**c**) Representative images of GFP^+^ cells (green) and HOECHST 33258 (blue) in contralateral and ipsilateral dorsal cortex over time. Scale bar=20 *μ*m. (**d**) Bar graphs of the quantitative analysis of the number of GFP^+^ cells in the selected ROIs of both hemispheres (*n*=3 mice for 1 week and 4 weeks; *n*=4 mice for 72 h and 2 weeks; *n*=5 mice for 8 weeks). Two-way ANOVA analysis demonstrates that both the ischemia (*P*<0.001) and time (*P*<0.05 for ventral cortex, *P*<0.01 for dorsal cortex and *P*<0.001 for corpus callosum and striatum) significantly affected the number of GFP^+^ cells. The effect of ischemia is reported in the small tables below each graph: *P<0.05, **P<0.01, ipsilateral *versus* contralateral side; Bonferroni post-hoc analysis. The effect of time is reported on each graph: *P<0.05, **P<0.01, ***P<0.001 *versus* 72 h; ^#^*P*<0.05, ^##^*P*<0.01, ^###^*P*<0.001 *versus* 1 week; ^$^*P*<0.05, ^$$^*P*<0.01, *versus* 2 weeks; Bonferroni post-hoc analysis

Figure 1a shows representative ipsilateral hemispheres 72 h, and 4 and 8 weeks after MCAo, immunostained with an anti-GFP antibody. GFP^+^ cells start accumulating early in the peri-infarcted area. With time, the ischemic area, as identified by MRI, became smaller and, 8 weeks after MCAo, GFP^+^ cells delimited this area. To detail this ischemia-induced cell reaction, we analyzed four different regions of interest (ROIs: dorsal cortex, ventral cortex, corpus callosum and striatum) (Figure 1b). Two-way ANOVA analysis demonstrates that both the *ischemia* and *time* factors significantly affected the number of GFP^+^ cells, but there was no interaction between these two parameters. Regarding the *ischemia* factor, at early time points (i.e., within 2 weeks) a significant increase in the number of GFP^+^ cells was observed in ventral and dorsal cortex of the ipsilateral compared with the contralateral side. At later time points (i.e., starting from 2 weeks), a significant increase in the number of GFP^+^ cells was observed only in corpus callosum and striatum. These different behaviors suggest a gradient in the detected changes, which develop with a slower kinetic in ROIs more distal from damage (Figures 1c and d). In sham-operated animals, no differences in the number of GFP^+^ cells in the ipsilateral compared with contralateral sides were found (data not shown). We conclude that the higher numbers of GFP^+^ cells in the ipsilateral side of MCAo mice are specifically related to induction of brain ischemia.

Regarding the *time* factor, an increase in GFP^+^ cells was observed ‘*per se*’ in the contralateral uninjured sides of all analyzed ROIs (Figures 1c and d), suggesting that the number of cells increases with age. In the ischemic sides at later times after MCAo, the number of GFP^+^ cells was significantly increased compared with 72 h only in corpus callosum and striatum (see legend of Figure 1d), suggesting different kinetics in the development of ischemic consequences in the different ROIs.

**Figure 2:**
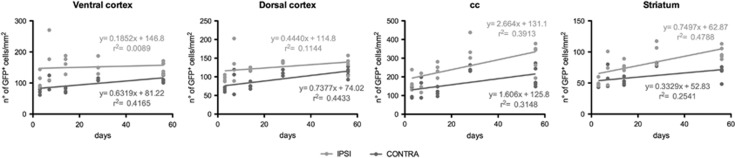
Correlation between the increase of GFP^+^ cells and days from the MCAo in the contralateral and ipsilateral ROIs. Scatter plot representation of the positive correlation between days from MCAo (*x* axis) and number of GFP^+^ cells (*y* axis) in different ROIs

To further investigate these differences, we then performed a Pearson and simple linear regression analysis (Figure 2). Data confirmed a positive correlation between the number of GFP^+^ cells and time from surgery in all contralateral uninjured ROIs, whereas in the ipsilateral ischemic sides this correlation was present only for striatum and corpus callosum (Supplementary Table 1). The lack of a linear correlation with time for ventral and dorsal cortex suggests that, in regions closer to damage, tissue remodeling is more complex and cannot be simply described by a linear equation. Data also suggest that, in these ROIs, changes are likely to have already occurred by 72 h.

### After MCAo, GFP^+^-OPCs are rapidly polarized toward the lesion and then undergo morphological changes

**Figure 3:**
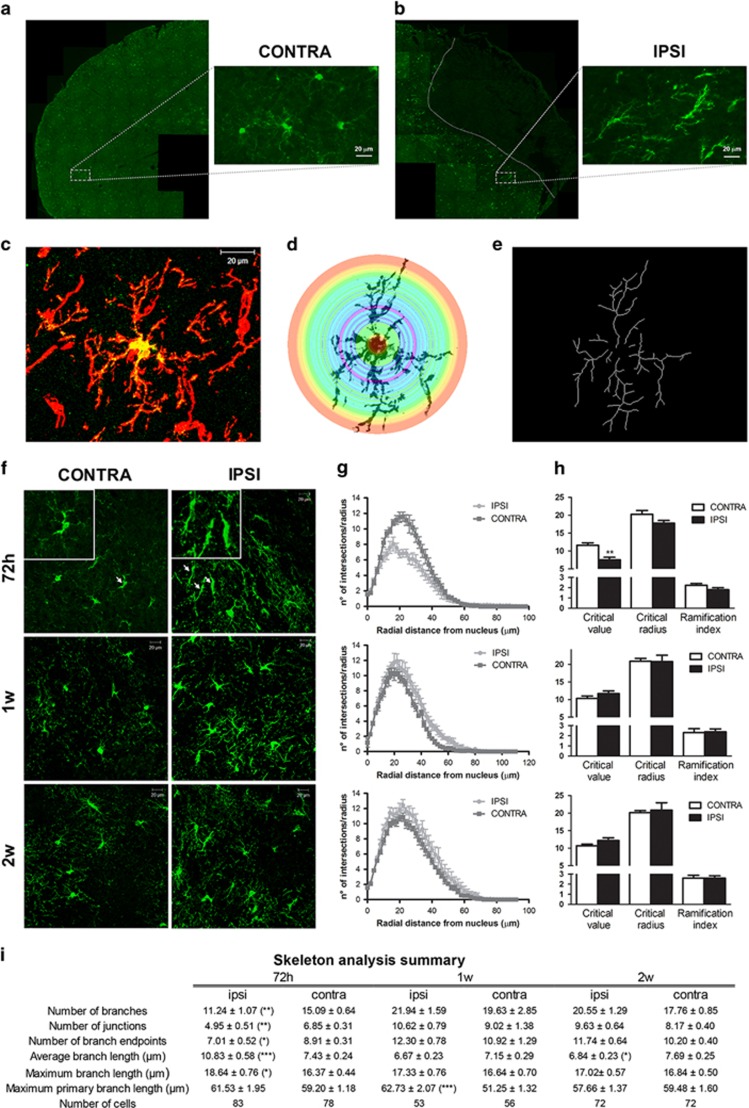
GFP^+^ cells undergo morphological changes in response to brain ischemia. (**a** and **b**) Representative images of GFP immunofluorescence of contralateral (**a**) and ipsilateral (**b**) hemispheres from GPR17iCreER^T2^:CAG-eGFP mice 72 h after MCAo. Insets show higher magnification of representative non polarized GFP^+^ cells with cell processes extending in all directions (contralateral side, **a**) or highly ‘polarized’ GFP^+^ cells showing a bipolar shape, with processes aligned with the cell body and extended toward the ischemic area (ipsilateral ischemic side in **b**; dotted line indicates the borders of the ischemic core). (**c**) Representative images of a GFP^+^ (green) and NG2^+^ (red) selected for the morphological analysis. Scale bar=20 *μ*m. (**d**) Illustration of Sholl analysis used to quantify process branching. The number of intersections made by the extending processes with each circle was automatic counted and used as a measure of process branching. (**e**) Illustration of skeletonized cells; Skeleton analysis was used to get information on the cell morphology complexity. (**f**) Representative images of GFP^+^ cells (green) and HOECHST 33258 (blue) in contralateral and ipsilateral dorsal cortex at 72 h, 1 week and 2 weeks after MCAo. High magnification of images at 72 h is reported. Scale bar=20 *μ*m. (**g**) Sholl analysis plot of GFP^+^ cells and (**h**) bar graphs of Critical value, Critical radius and Ramification index at 72 h, 1 week and 2 weeks after MCAo. (**i**) Skeleton analysis data summary. *P<0.05; **P<0.01; ***P<0.001; Student’s *t*-test

Close to ischemic damage, OPCs have been reported to assume monopolar/bipolar shape with elongated processes,^14^ which have been interpreted as a prerequisite for their migration.^15^ To investigate whether MCAo modifies the morphology of GFP^+^ cells, we performed a Sholl and Skeleton analysis in dorsal cortex regions at 72 h, 1 and 2 weeks after MCAo (Figures 3c–e).

At 72 h, in ipsilateral side, GFP^+^ cells showed a simpler morphology with fewer and more elongated processes polarized to the lesion, to suggest that these cells are attracted to the damaged area (Figures 3b and f). Indeed, ischemia induced an early change of the Sholl plot profile, a reduction of the critical value, a decrease of the number of branches, process endpoints and junctions, and an increase of branch length parameters (Figures 3g–i). On the contrary, 1 and 2 weeks, morphological parameters did not show any significant difference, suggesting that the morphological changes of GFP^+^ cells mainly occur at early times (Figures 3g–i).

To investigate whether the morphology of GFP^+^ cells was influenced by ischemia-induced maturation of OPCs, we analyzed separately the GFP^+^/NG2^+^ and GFP^+^/NG2^−^ cells (representing, respectively, early or more mature OPCs). We found that the morphological changes observed in GFP^+^ cells in ipsilateral side at 72 h, were restricted to the GFP^+^/NG2^+^ cellular sub-population. A detail description of the results is reported in Supplementary Figure 2.

Globally, these results indicate that ischemia induces phenotype- and time-dependent changes in the morphological structure of GFP^+^ cells.

### The migratory capacity of GFP^+^-OPCs is markedly increased after MCAo

**Figure 4:**
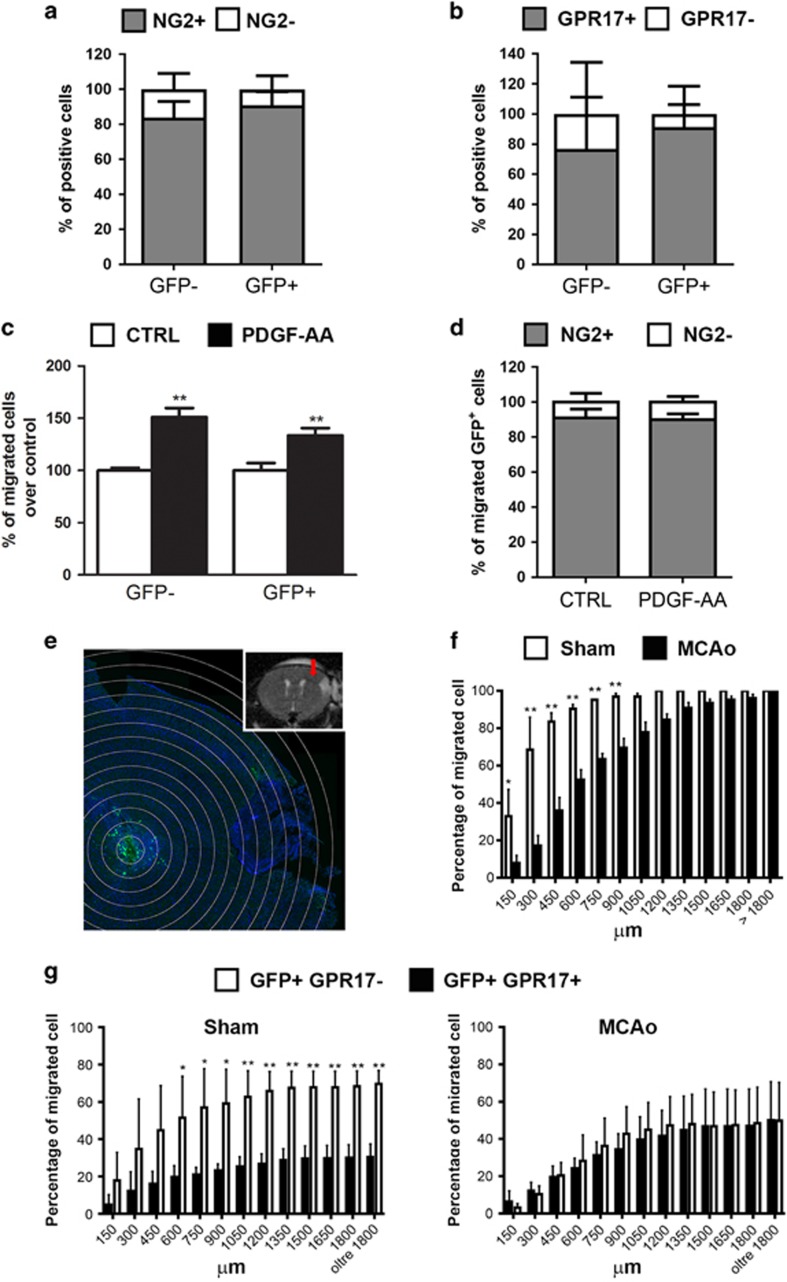
The migration capacity of GFP^+^ cells is influenced by brain ischemia. (**a** and **b**) Stacked bar graphs show the quantitative analysis of percentages of NG2^+^ and NG2^−^ cells (**a**) and of GPR17^+^ and GPR17^−^ cells (**b**) both within the GFP^+^ and GFP^−^ cell population (*n*=3 coverslips from three independent OPC preparations). (**c**) Bar graphs show the results of the quantitative analysis of cell migration assay performed in three independent experiments of GFP^+^ and GFP^−^ cells. Changes in number of migrated GFP^+^ cells were related to control condition set to 100%. **P<0.01; Mann–Whitney analysis. (**d**) Stacked bar graphs show the results of the quantitative analysis of percentages of NG2^+^ and NG2^−^ cells within the migrated GFP^+^ cell population (*n*=3 independent experiments). (**e**) Representative image of GFP immunohistochemistry of an ischemic wild-type mouse at 7 days after the injection. The concentric circles represent the distances from the injection site, starting from 150 *μ*m. In the insert is reported the T2W image of brain coronal section of the same mouse acquired 24 h after MCAo and before the injection of GFP^+^ OPCs. The red arrow represents the injection track. (**f**) The bar graph shows the quantification of the percentage of GFP^+^ cells, which have migrated at different distances from the injection site (*n*=2 sham-operated mice; *n*=3 MCAo mice). *P<0.05; **P<0.01; two-way ANOVA followed by Bonferroni post-hoc analysis. (**g**) The bar graph shows the quantification of the percentage of GFP^+^/GPR17^+^ and GFP^+^/GPR17^−^ cells, which have migrated at different distances from the injection site (*n*=2 sham-operated mice; *n*=3 MCAo mice). *P<0.05; **P<0.01; two-way ANOVA followed by Bonferroni post-hoc analysis

To assess whether the ischemia-induced morphological changes of GFP^+^-OPCs are indeed associated with altered migratory abilities, we performed an *in vitro* cell migration assay. For these experiments, we used OPCs isolated from neonatal GPR17iCreER^T2^:CAG-eGFP fluorescent mice containing both GFP^+^ and GFP^−^ cells. The vast majority of these GFP^+^ and GFP^−^ cells expressed both NG2 (90.89±4.48% and 83.86±5.29%, respectively; Figure 4a) and GPR17 (91.28±8.67% and 76.85±8.71%, respectively; Figure 4b). In the presence of PDGF-AA, a known chemoattractant factor accumulating at ischemic lesions, both GFP^+^ and GFP^−^-OPCs showed significantly increased motility (33.42±7.09% and 51.0±8.67%, respectively) compared with unstimulated cells (Figure 4c), to confirm that, in a similar way to normal OPCs, recombinant GFP^+^-OPCs are sensitive to chemoattractant signals.^16^

To evaluate whether the presence of NG2 modifies GFP^+^ cell migratory capacity, we better characterized the GFP^+^ migrated cell population. No differences in the percentage of NG2^+^ cells within GFP^+^ migrated cells were found either in the presence or in the absence of PDGF-AA. Moreover, we observed no significant changes in the percentage of NG2^+^ and NG2^−^ cells in the GFP^+^ cell populations before (Figure 4a) and after (Figure 4d) migration. Taken together, these data suggest that the presence of NG2 does not confer additional migratory capacities to the specific subcellular population of OPCs expressing GPR17 (Figure 4d).

To detail the migratory abilities of OPCs *in vivo* after MCAo, we transplanted fluorescent GFP^+^-OPCs in wild-type mice (Figure 4e). At the time of the graft, almost all fluorescent transplanted cells did express GPR17 (Figure 4b and above). One week after the graft, many GFP^+^-OPCs had moved away from the site of injection of a distance up to 900 *μ*m in MCAo mice showing greater migratory abilities compared with sham-operated mice (Figure 4f).

Then, to investigate whether the presence of GPR17 confers different abilities to the transplanted cells, we quantified the percentage of GFP^+^ cells expressing or not expressing GPR17 1 week after the injection. In sham-operated mice, the percentage of all the transplanted GFP^+^ cells that still maintained GPR17 was significantly lower (about 30%) compared with the cells that had lost the receptor (Figure 4g), suggesting progression to more differentiated stages. Instead, in MCAo mice, the percentage of cells maintaining GPR17 was about the 50% of total GFP^+^ cells (Figure 4g).

Altogether, these data demonstrate that the ischemic environment modifies the mobility of GFP^+^-OPCs, recruiting them from the parenchyma to the areas surrounding the lesion and likely inducing the release of factors maintaining GPR17 expression and thus preserving GFP^+^ cells at a precursor stage. We also speculate that the presence of GPR17 favors OPC migration, as shown by the increased number of GPR17^+^ cells at greater distances from the injection side in MCAo compared with sham-operated mice.

### After MCAo, GFP^+^-OPCs are also rapidly induced to proliferate

**Figure 5:**
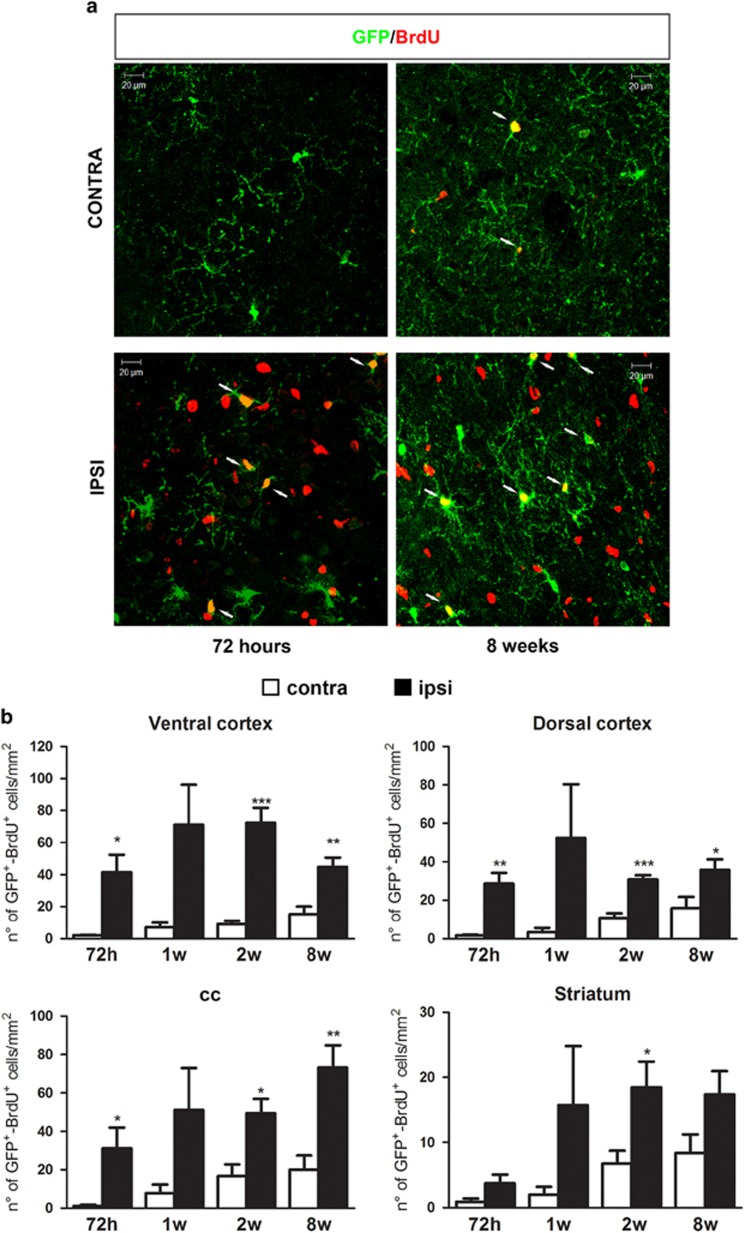
Brain ischemia induces proliferation of GFP^+^ cells. (**a**) Representative images of GFP (green) and BrdU (red) immunofluorescence in contralateral and ipsilateral dorsal cortex of mice 72 h and 8 weeks after MCAo. Arrows show double-positive cells. Scale bar=20 *μ*m. (**b**) Bar graphs showing the quantitative analysis of the number of BrdU^+^-GFP^+^ cells (*n*=3 mice for 1 week; *n*=4 mice for 72 h and 2 weeks; *n*=5 mice for 8 weeks). *P<0.05, **P<0.01, ***P<0.001 ipsilateral *versus* contralateral side; Student *t*-test. In contralateral dorsal cortex and striatum 2 weeks *versus* 72 h *P<0.05; one-way ANOVA followed by Bonferroni *post-hoc* analysis

To understand whether the MCAo-induced increase in the number of GFP^+^ cells could be because of a higher proliferation rate, we treated animals with 5-Bromo-2′-deoxyuridine (BrdU) (Supplementary Figure 1). We found that, in ipsilateral regions, the number of GFP^+^/BrdU^+^ cells increased with time (Figures 5a and b). Of note, not all GFP^+^ cells colocalized with BrdU, suggesting the presence of a sub-population of GFP^+^ cells that are more prone to proliferate after MCAo. As expected from previous data suggesting that OPCs do slowly, but significantly, proliferate under physiological conditions,^17^ an age-dependent increase in the number of proliferating GFP^+^ cells was also found in the unlesioned side (i.e., dorsal cortex: *P*<0.05, striatum: *P*<0.05; Figure 5b) and in sham-operated animals. However, in the latter, no statistically significant differences were detected between the ipsi- and contralateral sides at any of the analyzed time points (data not shown). Globally, these results show that, at least in part, the increased number of GFP^+^ cells can be ascribed to ischemia-induced higher proliferation rate.

### The final fate of the GFP^+^ cells is altered by brain ischemia

To better characterize the phenotypic changes of the GFP^+^ cells pool during the post-ischemic period, we performed a fate-mapping analysis with stage-specific oligodendrocyte markers (NG2 for early OPCs, GPR17 for immature oligodendrocytes and GSTpi for mature oligodendrocytes).

**Figure 6:**
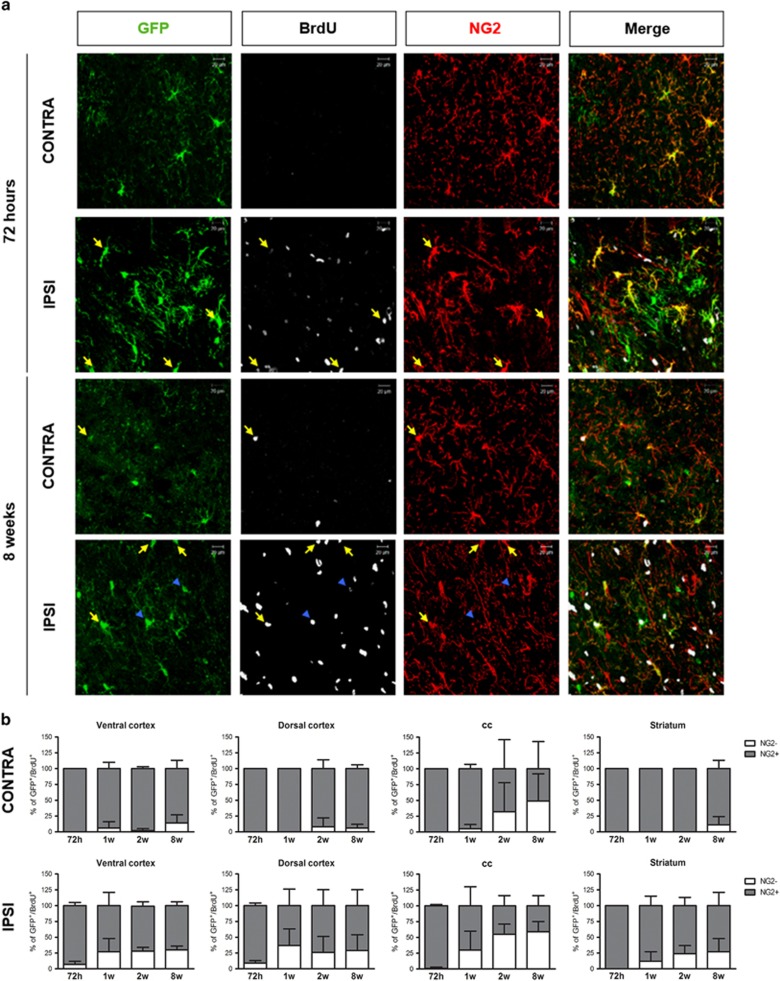
The fate of the GFP^+^/BrdU^+^ cells is altered by brain ischemia. (**a**) Representative images of GFP (green), BrdU (white) and NG2 (red) immunofluorescence in contralateral and ipsilateral dorsal cortex of mice 72 h and 8 weeks after MCAo. Arrows (yellow) show GFP^+^/BrdU^+^ cells positive for NG2 whereas head arrows (blue) show GFP^+^/BrdU^+^ cells negative for NG2. Scale bar=20 *μ*m. (**b**) Stacked bar graphs show the quantitative analysis of percentages of NG2^+^ and NG2^−^ cells within the GFP^+^/BrdU^+^ cell population in contralateral and ipsilateral ROIs (*n*=3 mice for 1 week and 8 weeks; *n*=4 mice for 72 h and 2 weeks) in the contralateral (upper graphs) and ipsilateral (lower graphs) side. NG2^+^ cells in contralateral ventral cortex: 8w *versus* 72 h *P<0.05; NG2^+^ cells in ipsilateral ventral cortex and corpus callosum: 8 weeks *versus* 72 h *P<0.05; NG2^−^ cells in ipsilateral corpus callosum and striatum: 8 weeks *versus* 72 h *P<0.05; one-way ANOVA followed by Bonferroni post-hoc analysis. NG2^+^ and NG2^−^ cells in ipsilateral striatum: *P<0.05; one-way ANOVA

As a first step, to get more information on the early proliferating GFP^+^/BrdU^+^ cells, we performed triple immunostainings with GFP, NG2 and BrdU antibodies (Figure 6a). At 72 h, almost all GFP^+^/BrdU^+^ cells still expressed NG2, indicating that during the acute reaction to injury all the proliferating recombined cells still remain at a precursor stage. Interestingly, already at 1 week, a percentage of these cells no longer expressed NG2. However, the loss of NG2 positivity reached statistical significance only at 8 weeks (i.e., *P*<0.05 in ventral cortex, corpus callosum and striatum; Figure 6b). In the contralateral side, negligible changes were observed (Figure 6b).

Our conclusion is that, after MCAo, GFP^+^ cells are initially induced to move toward the lesion while increasing their proliferation rate and that, subsequently, a subset of these cells is undergoing differentiation.

**Figure 7:**
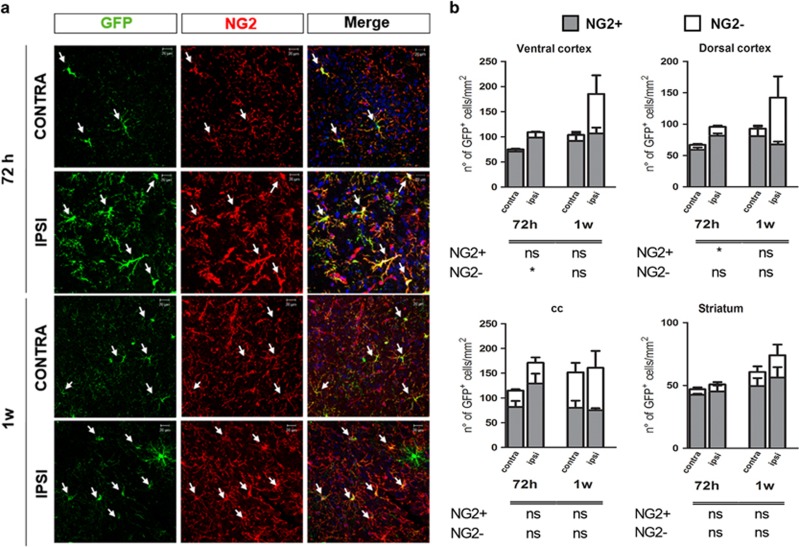
Early phase after MCAo, GFP^+^ cells mainly express the NG2 antigen. (**a**) Representative images of GFP (green) and NG2 (red) immunofluorescence in contralateral and ipsilateral dorsal cortex of mice 72 h and 1 weeks after MCAo. Arrows show GFP^+^/NG2^+^ cells. Scale bar=20 *μ*m. (**b**) Stacked bar graphs show the quantitative analysis of the number of GFP^+^/NG2^+^ cells (*n*=4 mice 72 h; *n*=3 mice for 1 week). *P<0.05 ipsilateral *versus* contralateral side; Student's *t*-test

**Figure 8:**
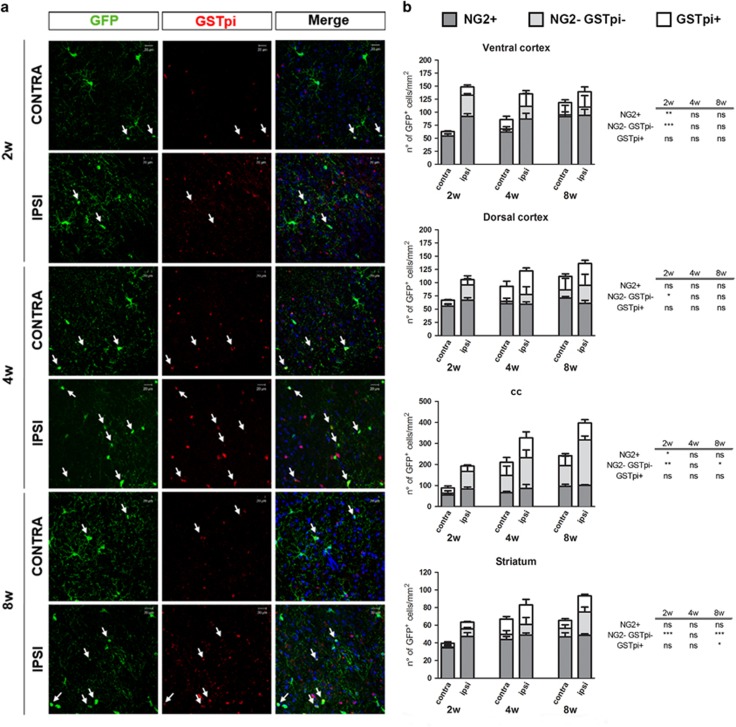
Late phase after MCAo, GFP^+^ cells progressively lose the NG2 antigen and start expressing the mature oligodendrocyte marker GSTpi. (**a**) Representative images of GFP (green) and GSTpi (red) immunofluorescence in contralateral and ipsilateral dorsal cortex of mice 2, 4 and 8 weeks after MCAo. Arrows show GFP^+^/GSTpi^+^ cells. Scale bar=20 *μ*m. (**b**) Stacked bar graphs show the quantitative analysis of the number of GFP^+^/NG2^+^ cells, GFP^+^/NG2^−^/GSTpi^−^ cells and GFP+/GSTpi^+^ cells (*n*=4 mice 2 weeks; *n*=3 mice for 4 weeks; *n*=5 mice for 8 weeks). *P<0.05, **P<0.01, ***P<0.001 ipsilateral *versus* contralateral side; Student's *t*-test

At 72 h, the number of GFP^+^/NG2^+^ cells was increased in the ipsilateral side in a different way according to the brain region, reaching statistical significance in dorsal cortex (*P*<0.05) (Figures 7a and b). This increase was still present at 2 weeks and then disappeared (Figure 8b), indicating that, after a very early recruitment of the NG2^+^/GFP^+^ cells, a pool of this OPC sub-population is preserved and does not undergo any further changes, to likely avoid exhaustion of brain’s repair abilities; conversely, a second pool of these recruited cells is addressed to maturation to sustain lesion repair.

Regarding this second pool of GFP^+^ cells, to discriminate cells at intermediate stages from more mature phenotypes, we analyzed in detail the following cell subpopulations: GFP^+^/GPR17^+^, GFP^+^/NG2^−^/GSTpi^−^ and GFP^+^/GSTpi^+^ cells. In line with our hypothesis, after MCAo, although less numerous compared with GFP^+^/NG2^+^ cells, GFP^+^/NG2^−^ cells initially expanded only in the ROIs closer to damage (*P*<0.05 in ventral cortex at 72 h); then, starting from 2 weeks, the number of GFP^+^/NG2^−^/GSTpi^−^ cells also significantly increased in corpus callosum and striatum, suggesting that ischemia is speeding up their maturation (Figures 7b and 8b). Moreover, in ventral cortex and striatum, a slight increase in GFP^+^/GPR17^+^ cells was observed at 8 weeks (Supplementary Figure 3). Globally, these data indicate that ischemia induces maturation of GFP^+^ cells.

As brain injury resulting in demyelination can induce adult OPCs to reacquire some stemcellness,^18^ we also tested the hypothesis that the GFP^+^/NG2^−^ sub-population of cells in the MCAo brain is not actually composed of more mature precursors, but indeed represent a new ischemia-induced population of cells reverting to more immature stages. We found that, up to 8 weeks after MCAo, no GFP^+^ cells expressed the stem cell marker GFAP (data not shown), suggesting that there is not a complete reversal to a stem cell stage. However, our data support recent results suggesting reversion to neonatal-like immature states of CNS progenitors characterized by migratory activity.^19^

Finally, quantitative analysis demonstrated that the number of GFP^+^/GSTpi^+^ cells slightly increased in all the considered regions, reaching a statistically significance at 8 weeks in striatum (*P*<0.05) (Figures 7b and 8a).

## Discussion

Not only neurons but also oligodendrocytes are severely affected by brain ischemia^20, 21^ and the loss of these cells causes myelin damage, contributing to stroke-associated deficits. Of note, the recovery of neurological function after stroke increases in parallel with white matter reorganization in rodents^22^ and microstructural integrity of white matter tracts is correlated with residual motor output in stroke patients.^23^ Thus, understanding the long-term behavior of OPCs, the myelin-producing cells, after stroke is crucial to find new strategies to promote endogenous remyelination and functional recovery.^24^

In this study, we have addressed this issue by using the GPR17iCreER^T2^:CAG-eGFP mice that allows to trace the proliferation, migratory abilities, differentiation and final fate of GPR17-expressing OPCs. As GPR17 is only transiently expressed by these precursors and completely disappears before they reach terminal maturation, these mice represent the only tool to follow the destiny of cells that have expressed GPR17.

We previously showed that, in this transgenic mouse line, GPR17^+^-OPCs (i.e., GFP^+^ cells) are reluctant to differentiate under healthy conditions, but can rapidly respond to injury, suggesting they may represent a ‘reserve pool’ of adult progenitors maintained for repair purposes.^10^ Here, we provide the first comprehensive *in vivo* fate-mapping analysis of these cells in a model of permanent brain ischemia up to 8 weeks.

In line with our previous results,^4, 12^ 72 h after MCAo, GPR17^+^ cells accumulate in the regions surrounding the lesions, and most of them still expressed the early OPC marker NG2. The increase of GFP^+^ cells persisted for at least 8 weeks with different regional kinetics that was faster in ROIs closer to the lesion. Highly relevant, a similar spatiotemporal gradient of the presence and activation of these cells has also been found in patients after traumatic brain injury.^8^

Our data show that the increased number of GFP^+^ cells around the ischemic lesion is, at least in part, because of ischemia-induced proliferation. The analysis of the number of GFP^+^ cells over time revealed that the increase of GFP^+^ cells mainly occurred in the early phases after MCAo. At later times, the rate of increase of these cells in the ipsilateral regions became similar to that observed in the contralateral ones, indicating a reduction with time of the ischemia-mediated effect. The proliferation rate of GFP^+^ cells indeed reached its maximum during the first week after MCAo, indicating that this pool of cells is extremely reactive to injury. Interestingly, some proliferating GFP^+^ cells were detected in the unlesioned side, and, consistent with previous data,^10^ a slower and more moderate increase of GFP^+^ cells was observed in sham-operated mice. This confirms that under physiological conditions, OPCs slowly but steadily proliferate throughout life.

Moreover, our data indicate that, besides proliferation, the increase of GFP^+^ cells is because of recruitment of migrating GFP^+^-OPCs toward the ischemic area. In this respect, GFP^+^-OPCs underwent early morphological changes, acquiring a simpler structure that can be also interpreted as an augmented migratory capacity. Specifically, 72 h after MCAo, the cellular complexity of GFP^+^/NG2^+^ cells significantly decreased in the ipsilateral side, and these cells exhibited fewer and more elongated processes in the direction of the lesion. Based on previous data showing that motile OPCs lose their un-oriented radial morphology and extend processes in the direction of migration^15^ and through the lesion,^25^ we conclude that the GFP^+^/NG2^+^ cells are indeed migrating toward the lesion. This conclusion is supported by our *in vitro* results, and by the increased migratory abilities of GFP^+^ cells when transplanted in MCAo mice.

These results are in line with our previous data demonstrating that GPR17 promotes OPCs migration *in vitro*^16^ and are consistent with literature results. Bipolar morphology was required for directional migration of OPCs *in vitro*, whereas increased number of cellular processes correlated with random and slow migration.^26^ In living brain slices, motile OPCs acquired a simple morphology whereas non-motile cells showed a complex morphology.^15^ These motile cells exhibited the same morphology described for OPCs moving from sites of proliferation to sites of injury after carotid ligation.^27^ As OPCs represent the majority of proliferating cells in the healthy adult brain,^17^ we do not rule out that some of the migrating GFP^+^/NG2^+^ cells may be newborn cells generated from the originally green-labeled GPR17^+^ cells.

The detected morphological changes weakened at later post-ischemia phases, when activated GFP^+^/NG2^+^ cells started maturating to myelinating phenotypes. In previous studies in healthy and stab wound injured brain, post-mitotic GPR17^+^ cells acquired a pre-myelinating phenotype. However, the exact relationship between proliferation and progression to mature oligodendrocytes remained uncertain,^5, 28^ and whether and how ischemia influenced the fate of these cells was unknown. The present data clearly show that, only starting from 2 weeks and to a greater extent 8 weeks after MCAo, GFP^+^-proliferating cells lose NG2 positivity, suggesting lineage progression to more advanced stages and terminal differentiation.

As OPCs of the gray and white matter are known to behave in different ways,^29^ to evaluate whether, depending on the extracellular microenvironment, the localization of the reacting GFP^+^ cells influences their differentiation properties, we detailed their behavior in either gray and white matter regions. Results indicate that the early recruitment of the GFP^+^/NG2^+^ cells in the regions surrounding the lesion was exclusively related to their proximity to the ischemic area.

The role of the expanded GFP^+^/NG2^+^ cells pool in the early phases after MCAo remains unclear. These cells may proliferate to assure that a sufficient number of cells undergoes myelination, and/or to assume completely different roles implementing brain repair. In this respect, it has been hypothesized that, beyond myelination, OPCs may also have trophic functions.^30^ OPCs indeed express high levels of active factors and cytokines regulating crucial biological processes like proliferation, differentiation and cell survival. In hippocampal slices undergoing oxygen glucose deprivation, release of BDNF and IL-10 by co-cultured OPCs improves cell survival and proliferation.^30^ The trophic effect of oligodendrocytes is not only restricted to OPCs but also extends to myelin, which sustains axonal homeostasis and neuronal survival.^31^ Transplanted human embryonic stem cells derived OPCs decrease infiltrating inflammatory cells within the subarachnoid space^32^ likely through increases of CCL2, CXCL10, sICAM-1 and TIMP-1, which, may, in turn, affect the migration of inflammatory cells.^32^ Another possible role of the increased GFP^+^/NG2^+^-pool could be to preserve the physical compartmentation of the brain. OPCs increase brain–blood barrier tightness via TGF-*β* signaling^33^ and may be also involved in scar formation. NG2-glia, together with microglia, are the first cells to react and strongly accumulate around the injury^34, 35^ and could be responsible for scar formation by building a high cell density area,^36^ as we also observed starting from 4 weeks after MCAo.

Notably, at later times after MCAo, the number of GFP^+^/NG2^+^ cells in the ipsilateral is similar to that of the contralateral side, indicating that, after ischemia-induced early activation, the pool of GFP^+^/NG2^+^ cells is restored to basal levels. Similarly, after brain stab wound, the number of NG2^+^ cells progressively decreases over time, leading to restoration of their physiological density.^10^ Globally, these data confirm that the adult brain does have the capacity to preserve the homeostasis of the OPC pool also in case of initial loss of oligodendrocytes, as observed in cerebral hypoperfusion.^37, 38^

Of note, the number of GFP^+^/NG2^-^/GSTpi^−^ cells, which likely are the pre-oligodendrocytes, is increased at 2 weeks after MCAo, suggesting that the initial activation of these cells is not finalized to promoting a rapid differentiation to myelinating phenotypes. In fact, the increase of percentage of GFP^+^ cells expressing GSTpi that reached a statistical significance only at later phases after MCAo.

In summary, this study characterizes the behavior and fate of GPR17-expressing OPCs in the ischemic brain over time. Results indicate that ischemia induces, sequentially: (i) early morphological changes of GPR17^+^ cells consistent with their migration toward the injury; (ii) a rapid proliferative response of GFP^+^ cells; (iii) a later increase of recombinant cells at intermediate stages of maturation; (iv) the subsequent progression of some of these cells to myelinating phenotypes.

Of interest, GPR17^+^ cells undergo maturation with different kinetics in the selected ROIs, depending upon their distance from the ischemic core, suggesting the existence of a gradient of factors that are released by the core area and act as differentiating agents, as nevertheless already suggested for GPR17^+^-OPCs in patients after traumatic brain injury.^8^ Moreover, the very early activation of these cells suggests that the role of OPCs goes well beyond myelination, indicating further un-explored trophic roles. The pharmacological manipulation of GPR17 will help establishing the ability of these cells to eventually sustain myelination and to improve the functional recovery of the regions surrounding the ischemic lesion.

## Materials and Methods

### Animals and experimental procedures

The procedures concerning animal care, surgery, killing were performed in accordance with national (D.L. n.26, 2014) and International laws and policies (EU Directive 2010/63/EU) and approved and authorized by the National Ministry of Health-University of Milan Committee (approval number 12/12-30012012 and 479/2015-PR). The protocol used is also in accordance with ARRIVE guidelines.

Eleven weeks old GPR17iCreER^T2^:CAG-eGFP report mice received 10 mg tamoxifen (Sigma-Aldrich, Taufkirchen, Germany), dissolved in 10% ethanol and 90% corn oil, three times *by gavage* once every second day. After 3 weeks of wash-out from tamoxifen, the mice were anesthetized with ketamine (80 mg/kg) and xylazine (16 mg/kg) and underwent permanent MCAo.^39^ Starting from 1 day before surgery, mice received 1 mg/ml BrdU (Sigma-Aldrich) in drinking water supplemented with 2% sucrose for 15 days. Mice were killed at 72 h and 1, 2, 4 and 8 weeks after MCAo (*n*=3–5 mice for each time point) (see Supplementary Figure 1). In parallel, sham-operated mice were included in each experimental group.

### Brain damage visualization

MRI investigations were performed in mice anesthetized with 1% isofluorane in 1l/min of O_2_ at 72 h and 1, 2, 4 and 8 weeks after ischemic insult using a 4.7 T, vertical super wide bore magnet of a Bruker Avance II spectrometer with micro imaging accessory (Ettlingen, Germany). Brain damage was visualized by means of T2-weighted MR imaging using a RARE sequence.^39^

### Immunofluorescence analysis

Mice were perfused with phosphate-buffered-saline and then 4% paraformaldehyde in phosphate-buffered-saline for at least 25 min. Brains were removed, post-fixed 1 h in the same solution and cryoprotected in 30% sucrose solution until precipitation at 4 °C. Coronal sections of 20-*μ*m were incubated with the following primary antibodies: chicken anti-GFP antibody (1:1400; Aves Labs, Inc., Tigard, OR, USA), rat anti-BrdU (1:150; Abcam, Cambridge, UK), rabbit anti-GSTpi (1:500; MBL, Woburn, MA, USA), rabbit anti-NG2 (1:2000; Millipore, Temecula, USA), rabbit anti-GFAP (1:300; Dako, Glostrup, Denmark) and rabbit anti-GPR17 (1:20 000; home-made antibody kind gift by P Rosa). Incubation with primary antibodies was made overnight at 4 °C in PBS with 1% normal goat serum (Dako) and 0.1% Triton-X 100, whereas for anti-NG2 in PBS with 1% normal goat serum and 0.3% Triton-X 100. The sections were exposed for 2 h at room temperature to secondary antibodies (Life Technologies, Monza, Italy). For rabbit anti-NG2 and GPR17, the signal intensity was enhanced using the High Sensitivity Tyramide-Rhodaminate Signal Amplification kit (Perkin-Elmer, Monza, Italy) following the manufacturer’s instructions. For triple GFP/NG2/BrdU labeling, staining of BrdU was performed last, after fixing sections with 4% paraformaldehyde for 10 min and incubating them with HCl 2N for 45 min at 37 °C. Nuclei were labeled with Hoechst 33258 (0.3 *μ*g/ml; Life Technologies).

### ROI selection and cell counts

The number of GFP-positive, double-positive GFP/BrdU, GFP/NG2, GFP/GPR17, GFP/GSTpi and triple-positive GFP/BrdU/NG2 was quantified, by a blinded investigator, on two slices per mouse from −1.00 to 0.00 mm from bregma.^40^ For the quantitative analysis, four anatomically defined ROIs surrounding the ischemic lesion, and their respective mirror ROIs in the contralateral side, were identified (ventral and dorsal cortex, corpus callosum and striatum). Briefly, after identifying the boundaries of the ischemic lesion we selected specific fields (281.25 × 281.25 *μ*m) for cell count as illustrated in the drawing (Figure 1b). Images were acquired at × 40 magnification using a confocal microscope (merge of 8-*μ*m z-stack at 2-*μ*m intervals; LSM510 META, Zeiss, Oberkochen, Germany).

### Sholl and skeleton analysis

A Sholl analysis method was developed to quantify GFP^+^, GFP^+^/NG2^+^ and GFP^+^/NG2^−^ cell morphology in immunofluorescent images of brain at 72 h, 1 and 2 weeks after MCAo. This morphological analysis was performed only with well-identified or isolated GFP^+^ cells, and on at least 18 cells for hemisphere of each mouse. Confocal images were acquired at each ipsilateral and contralateral dorsal cortex as detailed in drawing image (Figure 1b). The local contrast of GFP channel was enhanced and a basic grayscale morphologic erosion was performed to optimize the visualization of cellular processes. The resulting images were converted to a binary signal and analyzed using Simple Neurite Tracer plugin of the Fiji software, ImageJ(https://imagej.net/Fiji/Downloads). Cellular branches were manually traced and the center for the Sholl analysis was pointed at the centroid of the nucleus. Concentric circles were automatically drawn beginning at 2 *μ*m from the center and increasing 2 *μ*m with every circle.

The Sholl analysis plugin was then applied to all traced cells to collect data on the number of intersections between branches and each increasing circle to create a Sholl plot. For each mouse, a mean Sholl plot was generated. The parameters used for statistical analysis were: critical radius, critical value and ramification index.

The skeleton analysis plugin was applied in the same way and the parameters used for statistical analysis were: number of branches, number of branch endpoints and number of junctions, average and maximum branch length, and maximum primary branch length.

### OPC cultures

P0-P1 mice pups were subcutaneously injected with 30 ng tamoxifen to induce recombination and killed after 3 days. Cortices were removed, mechanically and enzymatically dissociated, suspended in Dulbecco’s modified Eagle's medium containing 10% fetal bovine serum, 4 mM l-glutamine, 1 mM Na-pyruvate, 100 U/ml penicillin, 100 U/ml streptomycin (all products from EuroClone, Milano, Italy), and plated in poly-l-lysin-coated 60 mm dishes (3 × 10^6^ cells). After 8 days, OPCs growing on top of a confluent monolayer of astrocytes were manually detached. Contaminating microglial cells were eliminated by plating detached cells on culture dishes for 1 h. OPCs were, then, collected and plated onto poly-dl-ornithine-coated dishes (Sigma-Aldrich) in Neurobasal medium (Life Technologies) containing 2% B27, 4 mM l-glutamine, 100 U/ml penicillin, 100 U/ml streptomycin, 10 ng/ ml platelet derived growth factor (PDGF-AA; Sigma-Aldrich) and 10 ng/ml basic fibroblast growth factor (bFGF; Space Import Export, Milan, Italy) to promote cell proliferation. After 2–3 days, cells were detached with accutase (Millipore) and used for migration assay or directly injected in wild-type mouse brain. Immunocytochemistry for GPR17 and NG2 was done to characterize the isolated OPCs.^3^

### Cell migration assay

Migration of GFP^+^-OPCs was performed with Boyden chambers (8 *μ*m pore size filter; Constar, Corning, NY, USA) as previously described.^16^ Briefly, the chamber was nested inside the well of 24-well plates and 5 × 10^4^ OPCs were seeded in the top of each insert with 200 *μ*l of neurobasal medium. The bottom well was filled with 600 *μ*l of medium containing the chemoattractant PDGF-AA (50 ng/ml). After 17 h, non-migrated cells were removed from the top compartment with a cotton swab, whereas cells that had migrated to the lower side of the filter were fixed with 4% paraformaldehyde and stained with Hoechst 33258, anti-GFP and anti-NG2 antibodies (see above). Images were acquired at × 20 magnification under an inverted fluorescence microscope (200M; Zeiss, Oberkochen, Germany) and cells counted using the Image J software (US National Institutes of Health, Bethesda, Marylan, USA). Data are expressed as a percentage of basal migration, that is, the migration of OPCs without chemoattractant.

### OPCs *in vivo* transplantation in MCAo mice

Wild-type mice (C57Bl/6J, 2- to 3-month old) underwent MCAo as described above. As a control, sham-operated mice were used. For cell transplantation, we followed a protocol previously described.^29^ One day after MCAo, 1 *μ*l of cell suspension (90 000 OPCs, see above) was injected in the ipsilateral striatum using a Hamilton syringe (Hamilton, 75 SN 5 *μ*l, Bonaduz, Switzerland). Coordinates of the site of injection were chosen based on the lesion area visualized by MRI (approximately +0.2 mm anterio-posterior, −2.0 mm medio-lateral, −3.0 mm dorso-ventral relative to the bregma). Mice were killed 1 week post-transplantation and the brains collected as described above. For each brain, we quantified the number of GFP^+^, GFP^+^-GPR17^+^ and GFP^+^-GPR17^−^ cells, which migrated from the injection site up to >1800 *μ*m in the surrounding tissue, and then calculated the percentage of moving cells. Cells remaining in the needle injection groove were not counted. We analyzed at least three slides for each mice and 2–3 animals per condition.

### Statistical analysis

The effect of time and ischemia on the number of GFP^+^ cells was evaluated by two-way ANOVA analysis followed by Bonferroni post-hoc analysis. The effect of time on proliferated GFP^+^ cells was evaluated by one-way ANOVA analysis followed by Bonferroni post-hoc analysis. The significance of the between-group differences was computed by means of unpaired Student’s *t*-test (ipsilateral *versus* contralateral side). Two-way ANOVA and Mann–Whitney analysis were used to detect statistical significance in migration assay. Pearson correlation test was used to evaluate the correlation between the number of GFP^+^ cells and the days from the surgery. Simple linear regression analysis was used to test whether the slopes and intercepts of the equation of a straight line obtained for contralateral and ipsilateral side were significantly different for each ROI. Data are expressed as mean±S.E.
