# Supplementary material for: The role of oligodendrocyte precursor cells expressing the GPR17 receptor in brain remodeling after stroke
Source: Cell Death Dis. 2017 Jun 8;8(6):e2871–. doi: 10.1038/cddis.2017.256 (PMC5520912; doi:10.1038/cddis.2017.256)
Supplement: Supplementary Information [file cddis2017256x1.docx]

The Supplementary Information file contains three supplementary figures (Supplementary Figure 1; Supplementary Figure 2; Supplementary Figure 3), their legends and one Table.

**Supplementary Information**

**Supplementary Figure 1. Experimental design.**

Eleven weeks old mice received tamoxifen by gavage three times and, after 3 weeks of wash-out, mice underwent permanent MCAo. Starting from 1 day before surgery, mice also received BrdU (1 mg/ml) in drinking water supplemented with 2% sucrose for 15 days. Mice were subjected to MRI investigations (T2W) at 24, 48 and 72h and at 1, 2, 4 and 8w after MCAo. Mice were sacrificed at 72h and 1, 2, 4 and 8w after surgery.

**Supplementary Figure 2. GFP+ cells undergo morphological changes in response to brain ischemia.**

**(A** and **B)** Sholl analysis plot and bar graphs of Critical value, Critical radius and Ramification index at 72h, 1w and 2w after MCAo of GFP^+^/NG2^+^ and GFP^+^/NG2^-^ cells. At 72 hours, GFP^+^/NG2^+^-cells in ipsilateral regions showed a simpler morphology, as demonstrated by their Sholl profile that was shifted to the left and down, in parallel to a significant decrease of the critical value. No changes in the profile of GFP^+^/NG2^+^-cells were observed at 1 and 2 weeks. In contrast, at 72 hours and 1 week, the Sholl profile of ipsilateral GFP^+^/NG2^-^-cells was shifted up, although the critical value was never statistically different. **(C** and **D)** Skeleton analysis data summary of GFP^+^/NG2^+^ and GFP^+^/NG2^-^ cells. The results of this analysis corroborated the data obtained with the Sholl analysis. At 72 hours, GFP^+^/NG2^+^ cells, but not GFP^+^/NG2^-^ cells, showed fewer branches, endpoints and junctions. Starting from 1 week, Skeleton analysis revealed a slight increase of process branching complexity in both cellular subpopulations.

**Supplementary Figure 3. The GPR17 expression in GFP^+^ cells is influenced by** **ischemia in region-dependent manner.**

Bar graphs show the quantitative analysis of the number of GFP^+^/GPR17^+^ cells (n=4 mice 2w; n=3 mice for 4w; n=5 mice for 8w). * *P* < 0.05, ** *P* < 0.01, *** *P* < 0.001 ipsilateral vs contralateral side; Student t-test.

**Supplementary Table 1.** Summary of Pearson and Linear Regression analysis for contralateral and ipsilateral side for each ROI.
