# Supplementary material for: The role of oligodendrocyte precursor cells expressing the GPR17 receptor in brain remodeling after stroke
Source: Cell Death Dis. 2017 Jun 8;8(6):e2871–. doi: 10.1038/cddis.2017.256 (PMC5520912; doi:10.1038/cddis.2017.256)
Supplement: Supplementary Table 1 [file cddis2017256x2.pdf]

Ventral cortex

	Pearson r	r^2	p	slope	intercept
CONTRA	0.6454	0.4165	0.0028	p=0.3847	p<0.0001
IPSI	0.0944	0.0089	0.7008		

Dorsal cortex

	Pearson r	r^2	p	slope	intercept
CONTRA	0.6658	0.4433	0.0019	p=0.421	p<0.0001
IPSI	0.3382	0.1144	0.1567		

Corpus callosum

	Pearson r	r^2	p	slope	intercept
CONTRA	0.5611	0.3148	0.0124	p=0.2927	p<0.001
IPSI	0.6255	0.3913	0.0042		

Striatum

	Pearson r	r^2	p	slope	intercept
CONTRA	0.5040	0.2541	0.0278	p=0.08484	p<0.001
IPSI	0.6919	0.4788	0.0010		
