# Supplementary figures and images for: The role of oligodendrocyte precursor cells expressing the GPR17 receptor in brain remodeling after stroke
Source: Cell Death Dis. 2017 Jun 8;8(6):e2871–. doi: 10.1038/cddis.2017.256 (PMC5520912; doi:10.1038/cddis.2017.256)

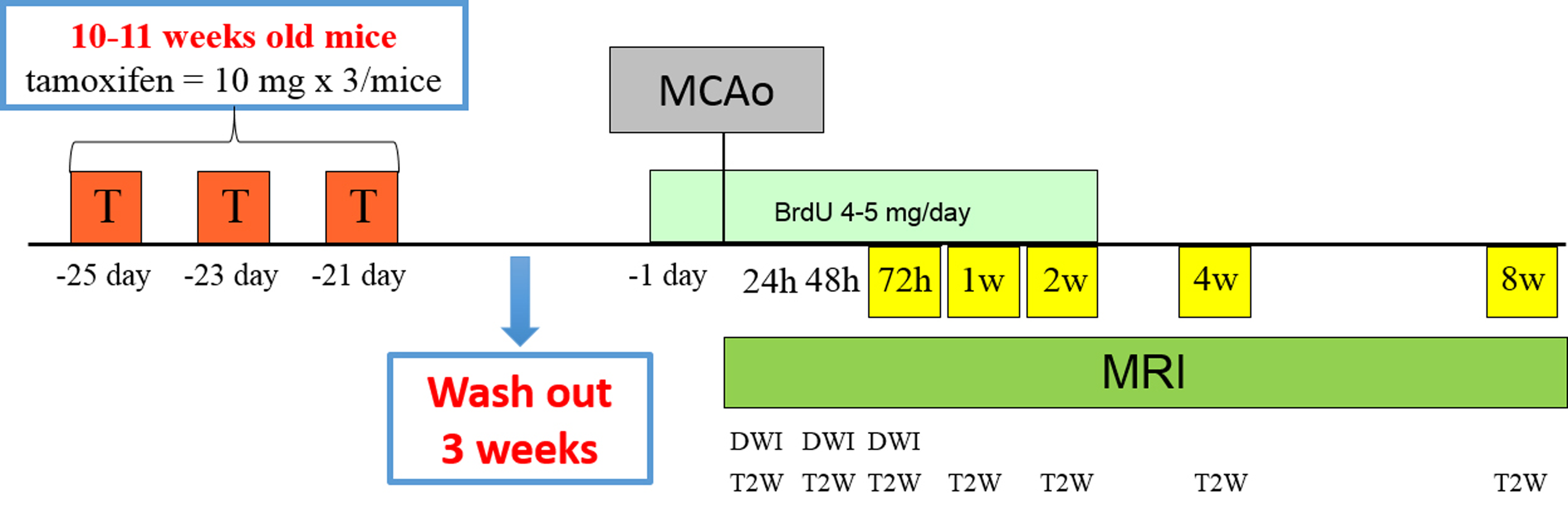

Supplement: Supplementary Figure 1 [file cddis2017256x3.tif]

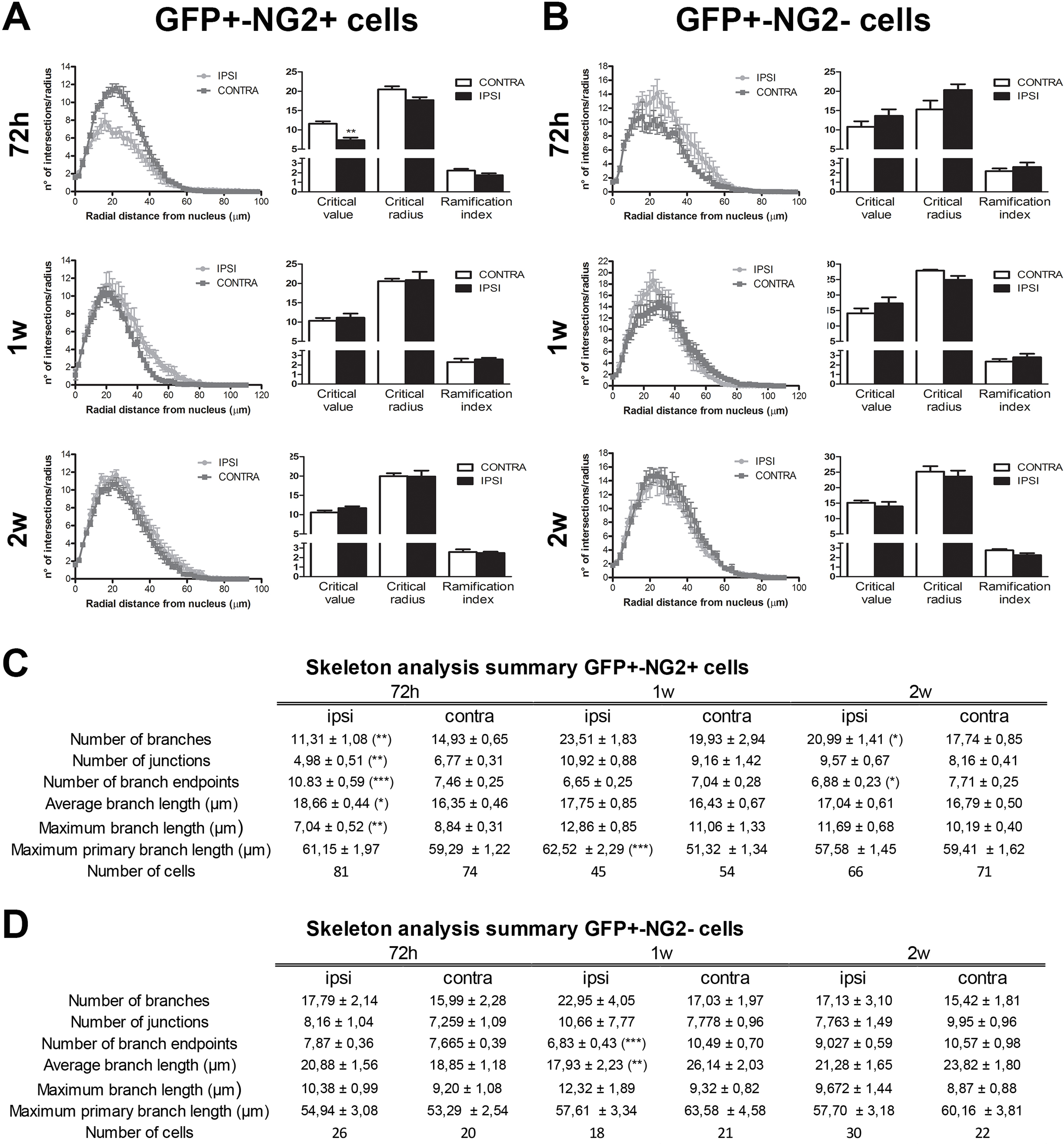

Supplement: Supplementary Figure 2 [file cddis2017256x4.tif]

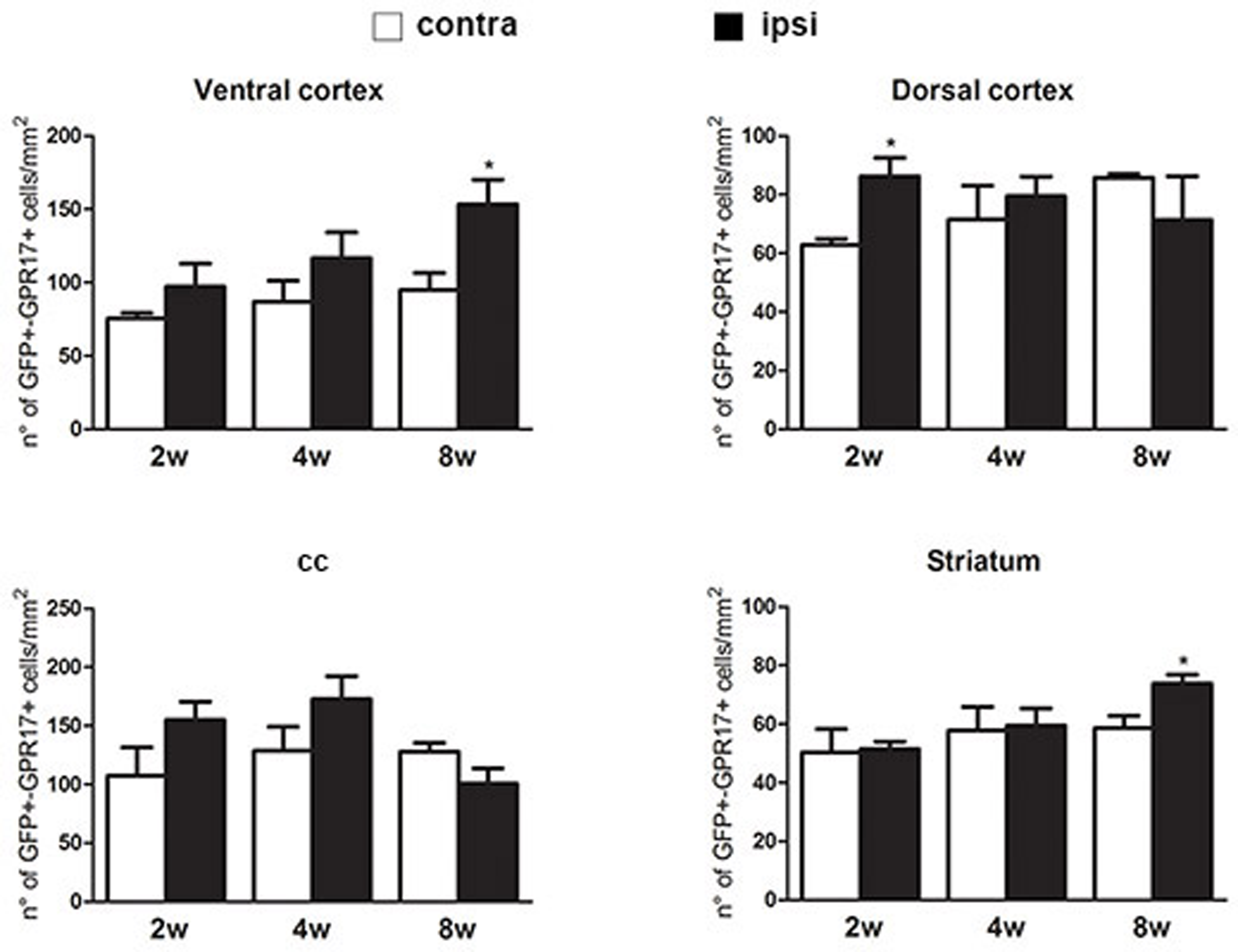

Supplement: Supplementary Figure 3 [file cddis2017256x5.tif]
